# Toward Fairness, Accountability, Transparency, and Ethics in AI for Social Media and Health Care: Scoping Review

**DOI:** 10.2196/50048

**Published:** 2024-04-03

**Authors:** Aditya Singhal, Nikita Neveditsin, Hasnaat Tanveer, Vijay Mago

**Affiliations:** 1 Department of Computer Science Lakehead University Thunder Bay, ON Canada; 2 Department of Mathematics and Computing Science Saint Mary's University Halifax, NS Canada; 3 Faculty of Mathematics University of Waterloo Waterloo, ON Canada; 4 School of Health Policy and Management York University Toronto, ON Canada

**Keywords:** fairness, accountability, transparency, and ethics, artificial intelligence, social media, health care

## Abstract

**Background:**

The use of social media for disseminating health care information has become increasingly prevalent, making the expanding role of artificial intelligence (AI) and machine learning in this process both significant and inevitable. This development raises numerous ethical concerns. This study explored the ethical use of AI and machine learning in the context of health care information on social media platforms (SMPs). It critically examined these technologies from the perspectives of fairness, accountability, transparency, and ethics (FATE), emphasizing computational and methodological approaches that ensure their responsible application.

**Objective:**

This study aims to identify, compare, and synthesize existing solutions that address the components of FATE in AI applications in health care on SMPs. Through an in-depth exploration of computational methods, approaches, and evaluation metrics used in various initiatives, we sought to elucidate the current state of the art and identify existing gaps. Furthermore, we assessed the strength of the evidence supporting each identified solution and discussed the implications of our findings for future research and practice. In doing so, we made a unique contribution to the field by highlighting areas that require further exploration and innovation.

**Methods:**

Our research methodology involved a comprehensive literature search across PubMed, Web of Science, and Google Scholar. We used strategic searches through specific filters to identify relevant research papers published since 2012 focusing on the intersection and union of different literature sets. The inclusion criteria were centered on studies that primarily addressed FATE in health care discussions on SMPs; those presenting empirical results; and those covering definitions, computational methods, approaches, and evaluation metrics.

**Results:**

Our findings present a nuanced breakdown of the FATE principles, aligning them where applicable with the American Medical Informatics Association ethical guidelines. By dividing these principles into dedicated sections, we detailed specific computational methods and conceptual approaches tailored to enforcing FATE in AI-driven health care on SMPs. This segmentation facilitated a deeper understanding of the intricate relationship among the FATE principles and highlighted the practical challenges encountered in their application. It underscored the pioneering contributions of our study to the discourse on ethical AI in health care on SMPs, emphasizing the complex interplay and the limitations faced in implementing these principles effectively.

**Conclusions:**

Despite the existence of diverse approaches and metrics to address FATE issues in AI for health care on SMPs, challenges persist. The application of these approaches often intersects with additional ethical considerations, occasionally leading to conflicts. Our review highlights the lack of a unified, comprehensive solution for fully and effectively integrating FATE principles in this domain. This gap necessitates careful consideration of the ethical trade-offs involved in deploying existing methods and underscores the need for ongoing research.

## Introduction

### Background

Machine learning (ML) algorithms have become pervasive in today’s world, influencing a wide range of fields, from governance and financial decision-making to medical diagnosis and security assessment. These technologies depend on artificial intelligence (AI) and ML to provide results, offering clear advantages in terms of speed and cost-effectiveness for businesses over time [1]. However, as AI research progresses rapidly, the importance of ensuring that its development and deployment adhere to ethical principles has become paramount.

User data on social media platforms (SMPs) can reveal patterns, trends, and behaviors. Platforms such as Twitter (X Corp) are predominantly used by younger individuals and those residing in urban areas [2]. These platforms often impose age restrictions, leading to a potential bias in algorithms trained on their data toward younger, urban demographics. Social media presents a rich source of data invaluable for health research [3], yet using these data without proper consent poses ethical concerns. Furthermore, social media content is influenced by various social factors and should not always be interpreted at face value. For example, certain topics may engage users from specific regions or demographic groups more than others [4], rendering the data less universally applicable. An additional challenge is the trustworthiness of these data. The issue of bias is further exacerbated when AI or ML software is proprietary with a closed source code, making it challenging to analyze and understand the reasons behind biased decisions [3].

The spread of both misinformation and disinformation is a significant concern on social media [5,6], a problem that became particularly acute during the COVID-19 pandemic. False claims about vaccine safety contributed to public mistrust and hesitancy, undermining efforts to control the virus. In tackling this issue, AI tools have been deployed to sift through information and spotlight reliable content for users [7]. These AI systems are trained using health data from trustworthy sources, ensuring the dissemination of scientifically sound information. On the bright side, social media provides a venue for disseminating new health information, offering valuable insights for the health sector [8]. However, the inherent challenges of social media, such as verifying information authenticity and the risk of spreading misinformation, require careful management to guarantee that the health information shared is accurate and reliable.

Fairness, accountability, transparency, and ethics (FATE) research focuses on evaluating the fairness and transparency of AI and ML models, developing accountability metrics, and designing ethical frameworks [9]. Incorporating a human in the loop is one approach to upholding ethical principles in algorithmic processes. For example, in the case of the Correctional Offender Management Profiling for Alternative Sanctions system used within the US judicial system to predict the likelihood of a prisoner reoffending after release, it is recommended that a judge first review the AI’s decision to ensure its accuracy. In summary, recognizing the inherent biases in AI and ML, the implementation of systematic models is crucial for maintaining accountability. Efforts in computer science are directed toward enhancing the transparency of AI and ML, which helps uncover the decision-making processes, identify biases, and hold systems accountable for failures [10,11].

### Motivation

The American Medical Informatics Association (AMIA) has delineated a comprehensive set of ethical principles for the governance of AI [12] building on the foundations laid out in the Belmont Report [13]: autonomy, beneficence, nonmaleficence, and justice. These principles are critical for the responsible application of AI in monitoring health care–related data on SMPs [7]. The AMIA expanded these principles to include 6 technical aspects—explainability, interpretability, fairness, dependability, auditability, and knowledge management—as well as 3 organizational principles: benevolence, transparency, and accountability. Furthermore, it incorporated special considerations for vulnerable populations, AI research, and user education [12]. Our review emphasized the concept of FATE, which is prevalent in the AI and ML community [14], and discussed its alignment with the principles outlined by the AMIA.

The discourse on AI ethics is notably influenced by geographic and socioeconomic contexts [15]. There has been extensive debate regarding the best practices for evaluating work produced by explanatory AI and conducting gap analyses on model interpretability in AI [16,17]. Recent advancements in ML interpretability have also been subject to review [18]. Table 1 provides a summary of existing studies that discuss FATE in various contexts. These studies reveal a substantial research gap in understanding how the principles of FATE are integrated within the realm of AI in health care on SMPs. Notably, none of the studies have thoroughly investigated the computational methods commonly used to assess the components of FATE and their intricate interrelationships in this domain.

To bridge the identified research gap, this study focused on three pivotal research questions (RQs):

1. What existing solutions address FATE in the context of health care on SMPs? (RQ 1)
2. How do these solutions identified in response to RQ 1 compare with each other in terms of computational methods, approaches, and evaluation metrics? (RQ 2)
3. What is the strength of the evidence supporting these various solutions? (RQ 3)

Our aim was to enrich the domain of FATE by exploring the array of techniques, methods, and solutions that facilitate social media interventions in health care settings while pinpointing gaps in the current body of literature. This study encompassed the definitions, computational methods, approaches, and evaluation metrics pertinent to FATE in AI along with an examination of FATE in data sets. The novelty of our research lies in delivering a comprehensive analysis of metrics, computational solutions, and the application of FATE principles specifically within the realm of SMPs. This includes a focus on uncovering further research directions and challenges at the confluence of health care, computer science, and social science.

**Table 1:** An overview of existing studies focusing on fairness, accountability, transparency, and ethics.

Study	Fairness				Accountability				Transparency				Ethics		
	A^a^	B^b^	C^c^	A		B	C	A		B	C	A		B	C
Mehrabi et al [1], 2021	✓	✓	✓												
Golder et al [19], 2017														✓	✓
Bear Don’t Walk et al [20], 2022	✓	✓	✓												
Attard-Frost et al [21], 2022	✓	✓	✓	✓		✓	✓	✓		✓	✓	✓			✓
Wieringa [9], 2020				✓		✓	✓								
Adadi and Berrada [22], 2018										✓	✓				
Diogo et al [18], 2019	✓						✓	✓		✓	✓				✓
Chakraborty et al [17], 2017	✓			✓				✓			✓				
Hagerty and Rubinov [15], 2019												✓			✓
Vian and Kohler [23], 2016				✓				✓							

^a^Definitions.

^b^Computational methods and approaches.

^c^Evaluation metrics.

## Methods

### Research Methodology

**Figure 1:**
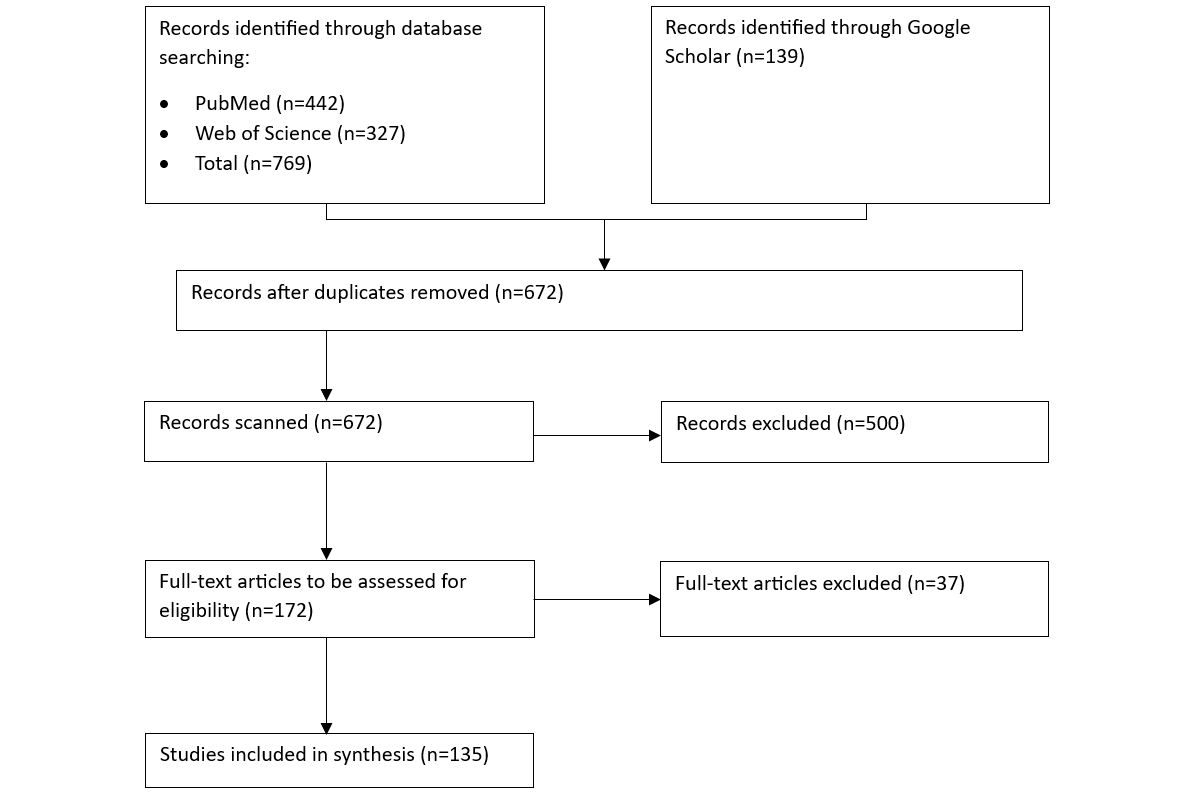
The PRISMA (Preferred Reporting Items for Systematic Reviews and Meta-Analyses) diagram for record selection.

Our research methodology was grounded in the approach presented by Kofod-Petersen [24] and adhered to the PRISMA-ScR (Preferred Reporting Items for Systematic Reviews and Meta-Analyses extension for Scoping Reviews) guidelines [25]. We used 2 search databases, PubMed and Web of Science, to ensure the reproducibility of the search results in the identification of records. PubMed was chosen for its comprehensive coverage of biomedical literature, providing direct access to the most recent research in health care and its intersections with AI, rendering it indispensable for studies focused on the FATE principles in the domain. Web of Science was selected for its interdisciplinary scope, diversity of publication sources, and rigorous citation analysis, offering a broad and authoritative overview of global research trends and impacts across computer science, social sciences, and health care. In addition, we used Google Scholar, which is recognized as the most comprehensive repository of scholarly articles [26], known for its inclusivity and extensive coverage across multiple disciplines. However, due to the lack of reproducibility of the search results on Google Scholar, we classified it as *other source* for record identification, as shown in Figure 1. Our search across these databases was conducted without any language restrictions, ensuring a comprehensive and inclusive review of the relevant literature.

We conducted a strategic search using Table 2 as a filter to identify research papers pertinent to our review. The table was designed to allow for customization of groups for retrieving varied sets of literature, aiming to find the intersection among these sets. For group 1, we selected “fairness,” “accountability,” “transparency,” and “ethics.” These keywords, being integral components of the FATE framework, were an obvious choice for our search queries. In group 2, we identified “natural language processing” and “artificial intelligence” as our keywords. The selection of “natural language processing” was justified by the predominance of textual data on SMPs, necessitating algorithms adept at processing natural language. The inclusion of “artificial intelligence” reflected its broad applicability beyond traditional ML applications. Given that AI encompasses a wide range of advanced technologies, including sophisticated natural language processing (NLP) techniques, its inclusion ensured the comprehensive coverage of relevant studies. Finally, the terms “social media” and “healthcare” were directly pertinent to our review, making their inclusion essential. Consequently, our aim was to encompass a wide spectrum of studies relevant to the topic of our review.

On the basis of Table 1, our initial strategy involved using the intersection of groups as follows: ([group 1, search term 1 ∩ group 2, search term 1] AND [group 1, search term 1 ∩ group 2, search term 2]) ∩ ([group 1, search term 1 ∩ group 3, search term 1] AND [group 1, search term 1 ∩ group 3, search term 2]), which, for simplicity, we condensed to (group 1, search term 1 ∩ group 2, search term 1 ∩ group 2, search term 2 ∩ group 3, search term 1 ∩ group 3, search term 2), as outlined in the search query presented in Textbox 1.

For our queries, we implemented year-based filtering in PubMed and conducted a parallel topic search in Web of Science, limiting the results to articles published since 2012. However, this approach yielded only 2 publications from each database, a tally considered inadequate for our purposes. Consequently, we opted to broaden our search by applying the union of 2 intersections. The initial formula ([group 1, search term 1 ∩ group 2, search term 1] AND [group 1, search term 1 ∩ group 2, search term 2]) ∪ ([group 1, search term 1 ∩ group 3, search term 1] AND [group 1, search term 1 ∩ group 3, search term 2]) was streamlined to group 1, search term 1 ∩ ([group 2, search term 1 ∩ group 2, search term 2] ∪ [group 3, search term 1 ∩ group 3, search term 2]), as detailed in the search query in Textbox 2, while maintaining the same year range.

Our search queries resulted in 442 records from PubMed and 327 records from Web of Science, as shown in Figure 1. Subsequently, we eliminated duplicates across the 3 sources, consolidating the findings into 672 records for initial screening. During the screening phase, we applied specific inclusion criteria based on an analysis of titles and abstracts to refine the selection: (1) the study primarily addressed FATE principles in the context of health care on SMPs (inclusion criterion 1); (2) the study reported empirical findings (inclusion criterion 2); (3) the study elaborated on definitions, computational methods, approaches, and evaluation metrics (inclusion criterion 3).

This process narrowed down the field to 172 records eligible for full-text assessment. At this stage, we applied our quality criteria to further assess eligibility: (1) we confirmed through full-text screening that the study adhered to inclusion criteria 1, 2, and 3 (quality criterion 1); (2) the study articulated a clear research objective (quality criterion 2).

Ultimately, this led to the selection of 135 articles for inclusion in our review. The complete list of these articles is available in Multimedia Appendix 1 [1-3,5-11,15-23,26-141].

**Table 2:** Search strategy for finding research articles.

	G1^a^	G2^b^	G3^c^
T1^d^	Quality^e^	Natural language processing	Social media
T2^f^	N/A^g^	Artificial intelligence	Health care

^a^G1: group 1.

^b^G2: group 2.

^c^G3: group 3.

^d^T1: search term 1.

^e^{Fairness, Accountability, Transparency, Ethics}

^f^T2: search term 2.

^g^N/A: not applicable.

**The initial query to the databases.**

- (“Fairness” OR “Accountability” OR “Transparency” OR “Ethics”) AND (“NLP” or “Natural Language Processing”) AND (“AI” OR “Artificial Intelligence”) AND (“Healthcare” AND “Social Media”)

**Modified query to the databases.**

- (“Fairness” OR “Accountability” OR “Transparency” OR “Ethics”) AND (((“NLP” or “Natural Language Processing”) AND (“AI” OR “Artificial Intelligence”)) OR (“Healthcare” AND “Social Media”))

### Data Items and Data-Charting

In our review, we incorporated the following data items: (1) approaches and definitions related to each component of FATE; (2) mathematical formulations and algorithms designed to address FATE; (3) methodologies for the integration of FATE principles into AI and ML systems, particularly within health care settings on SMPs; (4) characteristics of the AI or ML systems under study, encompassing their type, application areas within health care, and the specific roles that SMPs play in these systems; (5) outcomes from the formal evaluation or assessment of FATE aspects within the studies, such as their impact on decision-making processes; (6) challenges and barriers reported in the implementation of FATE principles in AI or ML systems; (7) use of frameworks or tools developed to support or evaluate FATE in AI and ML systems; and (8) engagement of stakeholders throughout the AI and ML system’s life cycle, including their perspectives on FATE.

The data-charting process involved 3 researchers, each independently extracting pertinent data from the selected sources with a particular focus on the aforementioned data items. For methodical organization and analysis, the extracted information was documented in Microsoft Excel spreadsheets (Microsoft Corp). These spreadsheets were organized alphabetically by the last name of the first author of each article and included references to the corresponding data items as presented in the studies. To consolidate the compiled data, one researcher was tasked with merging the information from these spreadsheets. This step aimed to synthesize the data and ensure a coherent presentation of our findings. The merging process entailed a thorough review and amalgamation of the data charted by each researcher, emphasizing the consolidation of similar approaches and methodologies as identified in the studies.

## Results

### Definitions, Computational Methods, and Approaches to Fairness

#### Overview

The understanding of fairness among the public is diverse [26]. The AMIA classifies fairness as a technical principle, emphasizing its importance in creating AI systems that are free from bias and discrimination [12]. This study reviewed various approaches to achieving fairness, with a particular focus on perspectives that facilitate the quantification of fairness in the context of AI for health care on SMPs. The mathematical formulations used to measure fairness are presented in Multimedia Appendix 2 [27-32,142,143]. The following subsections offer a comprehensive examination of approaches to ensure fairness.

#### Calibrated Fairness

Calibrated fairness seeks to balance providing equal opportunities for all individuals with accommodating their distinct differences and needs [33]. For instance, in the context of social media, a calibrated fair algorithm aims to ensure equal access to opportunities, such as visibility for all users, while also considering specific factors, such as language or location, to offer a personalized experience. In health care, such an algorithm would ensure that all patients have access to the same standard of care yet take into account variables such as age and health status to tailor the best possible treatment plan. The objective is to find a balance between treating everyone equally and acknowledging individual differences to achieve the most equitable outcomes. Fairness metrics, including the false positive rate difference [29] and the equal opportunity difference [34], are used to evaluate the degree of calibrated fairness. Common computational methods used to achieve calibrated fairness include the following: (1) preprocessing—modifying the original data set to diminish or eliminate the impact of sensitive attributes (eg, gender and ethnic background) on the outcome of an ML model [35]; (2) in-processing—integrating fairness constraints into the model’s training process to ensure calibration with respect to sensitive attributes [35]; (3) postprocessing—adjusting the model’s output after training to calibrate it in relation to sensitive attributes [35]; (3) adversarial training—training the model on adversarial examples, which are designed to test the model’s fairness in predictions [36].

Each of the approaches to achieving calibrated fairness in AI systems has a specific application context that is influenced by various factors. Preprocessing aims to directly mitigate biases in the data before the model’s training phase but may present challenges in preserving the integrity of the original data, potentially resulting in the loss of important information. In contrast, in-processing involves the integration of fairness constraints during the model’s learning process, which, while aiming to ensure fairness, might compromise model performance due to the added constraints. Postprocessing, which adjusts the model’s outputs after training, may appear as a straightforward solution but often falls short in addressing the root causes of bias, thus providing a superficial fix. Adversarial training stands out as a promising approach by challenging the model’s fairness through specially designed examples; however, its effective implementation can be complex and resource intensive. Each method has inherent trade-offs between fairness, accuracy, and complexity. The choice among them depends on the specific circumstances of the application, including the nature of the data, the criticality of the decision-making context, and the specific fairness objectives.

#### Statistical Fairness

Statistical fairness considers various factors, including demographic information, that may be pertinent to the concept of fairness within a specific context. Among the widely recognized statistical definitions of fairness are demographic parity, equal opportunity, and equal treatment [37]. The measure of “demographic parity” is used to reduce data bias by incorporating penalty functions into matrix-factorization objectives [38], whereas the “equal opportunity” metric is crucial for ensuring that decisions are devoid of bias [39]. In the realm of social media, individual notions of fairness might encompass issues such as unbiased content moderation, equitable representation of diverse perspectives and voices, and transparency in the algorithms used for content curation and ranking. Common approaches for measuring statistical fairness include the following: (1) equalized odds—this approach evaluates fairness by examining the differences in true positive and false positive rates across various groups [40]; (2) theorem of equal treatment—this approach assesses fairness by comparing how similar individuals from different groups are treated [41].

Moreover, several toolkits have been developed for measuring statistical fairness in ML and AI models. For instance, Aequitas, as introduced by Saleiro et al [42], generates reports aiding in equitable decision-making by policy makers and ML researchers. The AI Fairness 360 toolkit [43] provides metrics and algorithms designed to reduce statistical biases that lead to the unfair treatment of various groups by ML models [44]. Another toolkit, Fairlearn [45], offers algorithms aimed at addressing disparities in the treatment of different demographic groups by an ML model.

#### Intersectional Fairness

This approach integrates multiple intersecting identity facets, such as race, gender, and socioeconomic status, into decision-making processes concerning individuals [46]. Its objective is to guarantee equitable treatment for all stakeholders, recognizing that the confluence of these identities may exacerbate marginalization and discrimination. Within the realm of social media, an algorithm designed with intersectional fairness in mind ensures that content is neither recommended nor censored in a manner that is prejudiced against a user’s race, gender, or socioeconomic status. Similarly, in health care, an algorithm that incorporates intersectional fairness aims to prevent the disproportionate allocation of medical treatments and resources. Intersectional fairness can be operationalized using the worst-case disparity method, which involves evaluating each subgroup individually and comparing the best and worst outcomes to ascertain the precision of the fairness score. Subsequently, the ratio of the maximum to minimum scores is calculated, with a ratio nearing 1 indicating a more equitable outcome [46]. Other prevalent methods and strategies for achieving intersectional fairness include the following: (1) constraint-based methods—these are designed to honor specific fairness constraints, such as providing equal treatment to different groups identified by multiple attributes, through mathematical optimization [47]; (2) causal inference methods—these aim to ensure that the algorithm’s outputs are unbiased by examining the causal relationships between inputs and outputs [48]; (3) decision trees and rule-based systems—these are used to guarantee that the algorithm’s decisions are informed by relevant factors and free from bias [49].

Constraint-based methods are adept at enforcing predefined fairness goals; however, the complexity of defining and optimizing these goals poses a significant challenge. In contrast to constraint-based methods, causal inference methods do not necessitate predefined fairness constraints but require a thorough comprehension of the data at hand. Erroneous assumptions regarding causality can result in flawed assessments of fairness. Decision trees and rule-based systems, owing to their interpretability, facilitate the understanding of algorithmic decisions. However, their simplicity may be a limitation as they may not adequately address the complexities inherent in various data sets. To mitigate some of the discussed shortcomings, supervised ranking, unsupervised regression, and reinforcement in fairness evaluation can be approached through pairwise evaluation [50]. This technique involves assessing an AI model’s performance by comparing its outputs against a preselected set of input data pairs.

### Definitions, Computational Methods, and Approaches to Accountability

#### Overview

The AMIA considers accountability a fundamental organizational principle, stressing that organizations should bear the responsibility for continuously monitoring AI systems. This includes identifying, reporting, and managing potential risks. Furthermore, organizations are expected to implement strategies for risk mitigation and establish a system for the submission and resolution of complaints related to AI operations [12]. In the following subsections, we explore prevalent views on accountability within the ML and AI community. In addition, we provide summaries of the measurements for different accountability components as identified in the reviewed literature, which can be found in Multimedia Appendix 3 [51-54,144].

#### Legal Accountability

Legal accountability encompasses the obligations of entities involved in designing, developing, deploying, and using AI systems for health care purposes on social media [55]. This responsibility includes ensuring that AI systems are developed and used in compliance with relevant laws and regulations in addition to addressing any adverse effects or impacts that might arise from their use. Legal accountability also covers issues such as data protection and privacy along with the duty to prevent the use of AI systems for discriminatory or unethical purposes. Commonly used conceptual methods for achieving legal accountability include the following: (1) transparency—this method involves making AI systems transparent, ensuring that their decision-making processes are explainable and comprehensible [56] (there are existing frameworks designed to enhance transparency in the accountability of textual models [57]); (2) documentation—this involves maintaining detailed records of the systems’ design, development, and testing processes, as well as documenting the data used for training them [58] (an initiative toward accountability is the implementation of model cards, which are intended to outline an ML model’s limitations and disclose any biases that it may be susceptible to [59]); (3) adjudication—this refers to the creation of procedures for addressing disputes and grievances associated with the use of ML and AI systems [60].

Overall, the pursuit of legal accountability should be carefully balanced with the autonomy of stakeholders and must not hinder innovation.

#### Ethical Accountability

Ethical accountability ensures that AI systems make decisions that are transparent, justifiable, and aligned with societal values [61]. This encompasses addressing data privacy, securing informed consent, and preventing the perpetuation of existing biases and discrimination. Ethical concerns specific to the use of AI in health care include safeguarding patient privacy, handling sensitive health data responsibly, and avoiding the reinforcement of existing health disparities [62]. Common strategies for achieving ethical accountability include the following: (1) ethical impact assessment—this approach entails assessing the ethical risks and benefits of the system and weighing the trade-offs between them [63]; (2) value alignment—this strategy involves embedding ethical principles and values into the design and development of the system, ensuring that its operations are in harmony with these values [64]; (3) transparency and explanation—this is accomplished by offering clear, understandable explanations of the system’s functionality and making its data and algorithms openly available [65]; (4) stakeholder engagement—this involves the active participation of a diverse group of stakeholders, including users, developers, and experts, in all phases of the AI or ML system’s life cycle [66].

When crafting ethical AI for disseminating health care–related information on social media, the application of these methodologies varies according to specific tasks. Ethical impact assessments, for instance, are valuable for evaluating the potential advantages, such as enhanced patient engagement via personalized dissemination of health care information, against risks, including privacy breaches and the spread of misinformation. The value alignment method plays a crucial role in pinpointing essential ethical values such as patient privacy, information accuracy, nondiscrimination, and accessibility. This method also supports the performance of regular audits to verify that AI systems continuously reflect these ethical standards. Finally, approaches to stakeholder engagement establish a platform for transparent and continuous communication between stakeholders and developers, thereby promoting a cooperative atmosphere in development.

#### Technical Accountability

Technical accountability ensures that developers and designers of AI and ML systems are held responsible for maintaining standards of security, privacy, and functionality [67]. This responsibility encompasses the implementation of adequate mechanisms to monitor and manage AI algorithms and address arising technical issues. Within the realms of social media and health care, technical accountability further entails the use of AI technologies to foster ethical decision-making, safeguard user privacy, and ensure that decisions are made fairly and transparently [68]. Common strategies for achieving technical accountability include the following: (1) logging—the practice of recording all inputs, outputs, and decisions to trace the system’s performance and pinpoint potential problems [69]; (2) auditing—conducting evaluations to check the system’s performance, detect biases, and ensure compliance with ethical and legal standards [70].

Both logging and auditing play critical roles in the development of ethical AI for health care information on social media, each with its unique benefits and challenges. Logging, which captures the inputs, outputs, and decisions of an AI system, is vital for tracking system performance. Nonetheless, the retention of detailed logs, especially those involving sensitive health care information, may introduce privacy concerns and necessitate careful consideration of data protection strategies. Auditing, essential for upholding ethical and legal norms, demands expertise and considerable time to effectively scrutinize complex AI systems. In addition, frameworks designed to enhance AI system accountability are in use. An example is Pandora [71], representing a significant move toward achieving a holistic approach to accountable AI systems.

#### Societal Accountability

Societal accountability entails the obligation of stakeholders to ensure that their AI systems align with societal values and interests [72]. This encompasses addressing privacy, transparency, and fairness issues, along with considering the wider social, cultural, and economic impacts that AI systems may have. Achieving societal accountability may require stakeholders to participate in public consultations, develop ethical and transparent regulations and standards for AI use, and enhance public understanding of AI system functionalities and applications. Essentially, it advocates for the development and use of AI systems under the principles of responsible innovation, with society’s interests considered at every life cycle stage.

Methods for ensuring societal accountability include the following: (1) regulation and standardization—creating regulations and standards for AI system design and use can help hold these systems accountable to society, safeguarding the rights and interests of all stakeholders [73]; (2) public-private partnerships—fostering collaboration among government agencies, private-sector companies, and other entities to promote the societal accountability of AI and ML systems [74].

To ensure accountability, integrating transparency and fairness into algorithms, designing systems with privacy considerations, and conducting regular audits and evaluations to review AI system performance is critical. Researchers have suggested approaches for holding companies accountable for their AI-related actions [9]. They emphasize the importance of pinpointing specific decision makers within a company responsible for any errors, a crucial step for ensuring equitable accountability. The entity or individuals determining accountability should possess comprehensive knowledge of legal, political, administrative, professional, and social viewpoints regarding the error to guarantee fair and unbiased judgments. Moreover, the consequences imposed on decision makers should be appropriately matched to their areas of responsibility, considering each individual’s level of responsibility within the company’s hierarchy when deciding on these consequences.

### Definitions, Computational Methods, and Approaches to Transparency

#### Overview

According to the AMIA, transparency is an organizational principle that asserts that an AI system must operate impartially, not favoring its host organization. This principle ensures fairness, treating all stakeholders equally without privileging any party. Moreover, transparency requires stakeholders to be clearly informed that they are interacting with an AI system and not a human [12]. Adadi and Berrada [22] presented a nuanced view on transparency, defining it as the degree to which the workings of an AI system are comprehensible to humans. This definition encompasses providing explanations for the system’s decision-making processes, clarifying the data used for system training, and certifying the system’s neutrality and nondiscriminatory nature. The balancing act between transparency and privacy presents challenges. For instance, in the analysis of mental health data on SMPs, the difficulty does not lie in pinpointing user-specific attributes (as data are often aggregated) but in the application of these data [75]. Here, transparency intersects with the ethical principle of autonomy, which demands that systems protect individual independence, treat users respectfully, and secure informed consent [12]. Guaranteeing autonomy is particularly crucial in the deployment of AI-powered depression detection systems on social networks [76]. The following subsections will delve into the nuances of transparency in AI, emphasizing the importance of openness in data and algorithmic procedures. This focus is particularly critical in the context of data derived from SMPs. We also introduce some metrics for assessing transparency in Multimedia Appendix 4 [77-81].

#### Algorithmic Transparency

Algorithmic transparency is the clarity with which one can comprehend the manner in which an AI algorithm or model produces its outputs or decisions [82]. Within the context of AI for health care on SMPs, transparency entails the ability to lucidly grasp the processes and methodologies used in the creation, dissemination, and evaluation of social media interventions for health care objectives [83]. This encompasses an understanding of the data sources that inform these interventions, the algorithms or models that analyze the data and generate the interventions, and the criteria for assessing intervention effectiveness. Algorithmic transparency is crucial for identifying and addressing potential biases or errors in interventions and fostering trust among stakeholders, including patients, health care providers, and regulatory bodies. Several computational techniques can enhance algorithmic transparency: (1) feature importance analysis—this technique identifies the most impactful features or variables in the model’s output, shedding light on the decision-making process [84]; (2) model interpretability—this involves designing models whose outputs are easily understood and interpreted by humans [85] (for instance, decision trees and logistic regression models are more interpretable compared to more complex models [86]; detailed discussions of model interpretability will follow in a dedicated subsection); (3) explanation generation—this technique produces explanations for a model’s outputs, offering insights into its decision-making process through visualizations or natural language descriptions [87].

Feature importance analysis enhances the comprehension of a model’s decision-making process, yet it may not fully elucidate the complex interactions among features or their combined effect on the model’s decisions, especially in the case of sophisticated deep neural networks. Models that are inherently interpretable, such as decision trees and logistic regression, promote user trust and facilitate the validation of model behaviors. However, these models might not offer the same level of power and precision as more complex models such as deep neural networks, which restricts their effectiveness in analyzing health care–related social media interactions. On the other hand, explanation generation seeks to clarify the model’s reasoning for stakeholders. Nonetheless, guaranteeing that these explanations are both accurate and reflective of the model’s inner workings poses a considerable challenge.

#### Data Transparency

Data transparency pertains to the comprehensibility of how data are collected, stored, and used in the development of an AI system [88]. Within the realm of AI for health care on SMPs, data transparency delineates the degree to which health care organizations and providers maintain openness and clarity regarding the collection, storage, and use of patient data [89]. This aspect is critical to the design and implementation of social media campaigns, encompassing the provision of explicit information to patients about the nature of the data being collected, their intended uses, the entities granted access, and the measures in place for their protection. By adopting a transparent approach to data collection and use, health care organizations can foster trust among patients and encourage more robust engagement in social media–driven health interventions. Such transparency can significantly enhance patient health outcomes as individuals are more inclined to engage in interventions in which they feel informed, comfortable, and confident. Examples of computational methods to enhance data transparency include the following: (1) data visualization—this method entails the creation of graphical representations of data to simplify user understanding and interpretation [90]; (2) data profiling—this process analyzes data to ascertain their structure, quality, and content, aiding in the identification of issues such as missing values and inconsistencies [91]; (3) data lineage analysis and provenance tracking—this approach tracks the movement of data through various systems and processes to verify their accuracy and reliability [81,92].

A critical consideration in implementing any of the data transparency methods is ensuring that the autonomy and privacy of all stakeholders are upheld.

#### Process Transparency

Process transparency denotes the capability to comprehend the procedures involved in the development and deployment of an AI system, including the testing and validation methodologies used [93]. Within the sphere of social media and health care, this notion extends to the clarity of decision-making processes that govern the prioritization, display, and dissemination of health-related information on SMPs. This encompasses transparency regarding the algorithms and computational methods used to curate and showcase health-related content as well as the policies and guidelines governing the moderation of user-generated content pertaining to health. Enhancing process transparency allows users to place greater trust in the information and interventions presented to them and affords researchers increased confidence in the data they examine. Several computational techniques can facilitate enhanced process transparency in AI systems: (1) auditability and monitoring—this involves integrating auditing and monitoring functions within the AI system, including tracking the system’s performance, detecting biases or other ethical concerns, and pinpointing instances of underperformance [94]; (2) open-source development—this entails the open and transparent creation of AI systems, where the code, data, and models are made accessible to the public. Such transparency fosters enhanced scrutiny and accountability of the system by external parties, including regulators and the general public [95].

Adopting these methods while recognizing their limitations and taking into account additional ethical considerations can foster greater transparency in AI applications for health care interventions on SMPs.

#### Explainability and Interpretability

According to the AMIA, the concepts of explainability and interpretability in AI are closely intertwined in the context of transparency. Explainability necessitates that AI developers articulate the functions of AI systems using language appropriate to the context, ensuring that users have a clear understanding of the system’s intended use, scope, and limitations. Conversely, interpretability concentrates on the system’s capability to elucidate its decision-making processes [12]. It is common for researchers to use the terms explainability and interpretability interchangeably [18,96].

In the realm of social media interventions for health care, explainability and interpretability pertain to comprehending how an AI system processes social media data, identifies pertinent information, and bases its recommendations or decisions on those data [97]. Research conducted by Amann et al [98] delves into the explainability aspects of AI in health care from 4 perspectives: technological, medical, legal, and that of the patient. The authors highlighted the critical role of explainability in the medical domain, arguing that its absence could compromise fundamental ethical values in medicine and public health. The pursuit of explainability and interpretability in AI systems remains a vibrant area of research. For AI systems that apply social media interventions in health care, various methods, including feature selection techniques and visualizations, can facilitate a deeper understanding among health care professionals of the AI system’s underlying mechanisms and the factors influencing its decision-making process. As Barredo Arrieta et al [99] noted, techniques for interpretability in AI involve the design of models with clear and comprehensible features, which can aid in identifying the factors that impact the AI’s decisions, thus simplifying the understanding and explanation of the outcomes. The existing computational approaches to achieving explainability and interpretability include the following: (1) partial dependence plots (PDPs) [98,100]—PDPs elucidate the relationship between specific input variables and the predicted outcome, offering insights into the rationale behind an AI model’s decisions; (2) local interpretable model-agnostic explanations (LIME)—LIME elucidates the outputs of ML models by creating a simpler, interpretable model that approximates the behavior of the original model [101]; (3) Shapley additive explanations (SHAP)—unlike LIME, SHAP explains the outputs of ML models by calculating the contribution of each input feature to the final output [102]; (4) counterfactual explanations—this approach identifies the minimal changes required in the input features to alter the model’s output, providing insights into alternative decision pathways [103]; (5) using mathematical structures for analyzing ML model parameters—techniques such as concept activation vectors, t-distributed stochastic neighbor embedding, and singular vector canonical correlation analysis are used for this purpose [104]; (6) attention visualization [105]—techniques for visualizing attention in transformer-based language models used across various NLP tasks on SMPs help reveal the models’ inner workings and potential biases; (7) explanation generation—this involves creating natural language or visual explanations for an AI system’s decisions (using techniques such as saliency maps, LIME [101], and SHAP [102] in conjunction with NLP methods enhances the generation of comprehensible explanations); (8) applying inherently interpretable models—models such as fuzzy decision trees, which graphically depict the decision-making process akin to standard decision trees, clarify how decisions are made and identify the most influential factors [106]; (9) model distillation—this technique trains a simpler model to approximate the decision boundaries of a more complex model, thereby facilitating the creation of an interpretable model while maintaining the original’s performance [107].

While all the aforementioned methods significantly contribute to the explainability and interpretability of AI and ML systems in this domain, it is crucial to recognize their inherent limitations in practical applications. Specifically, PDPs may face challenges with complex unstructured data such as natural language. SHAP can become computationally intensive when dealing with a large number of input features, which is typical in complex models. LIME might yield inconsistent outcomes, and the interpretations from attention visualization techniques necessitate detailed analysis by experts. Explanation generation, which is often dependent on the aforementioned methods, can inherit their flaws, potentially resulting in misleading explanations. Finally, models that are inherently interpretable or refined through distillation techniques might oversimplify, failing to fully encapsulate the complexities of health care interventions on SMPs.

### Definitions, Computational Methods, and Approaches to Ethics

#### Overview

Ethics encompasses a wide range of considerations, many of which align with the AI principles recognized by the AMIA. In the realm of AI, ethics generally pertains to the study and practice of crafting and applying AI technologies in ways that are fair, transparent, and advantageous to all stakeholders [108]. The objective of ethical AI is to ensure that AI systems and their decisions are in harmony with human values, uphold fundamental human rights, and do not cause harm or discrimination to individuals or groups. This encompasses issues related to privacy, data protection, bias, accountability, and explainability [109].

Within the sphere of social media, the digital surveillance of public health data from SMPs should adhere to several key principles: (1) beneficence, ensuring that surveillance contributes to better public health outcomes; (2) nonmaleficence, ensuring that the use of data does not undermine public trust; (3) autonomy, either through the informed consent of users or by anonymizing personal details; (4) equity, ensuring equal access for individuals to public health interventions; and (5) efficiency, advocating for legal frameworks that guarantee continuous access to web platforms and the algorithms that guide decision-making [110]. AI-mediated health care interventions must consider affordability and equity across the wider population. In addition, health-related data gathered from social platforms need to be scrutinized for various biases such as population and behavioral biases using appropriate metrics [111]. The following subsections offer insights into different ethical viewpoints and the methods used to evaluate how well AI systems align with these ethical standards. We also present summaries of quantifications of key ethical elements in Multimedia Appendix 5 [112-115].

#### Philosophical Ethics

Our review concentrated primarily on the practical application of ethical principles in AI rather than exploring the purely philosophical dimensions of ethics. Consequently, this subsection focuses on a set of general ethical principles directly pertinent to AI. Kazim and Koshiyama [116] examined various philosophical aspects of ethics and supported a human-centric approach to AI. This perspective underscores the significance of designing and using AI systems in ways that uphold human autonomy, dignity, and privacy [116]. Within the realm of health care interventions on social media, the philosophical ethics of AI can be specifically perceived as the application of ethical principles and values to the development and use of AI-powered tools and technologies [117]. This entails scrutinizing the potential benefits and risks associated with using AI to gather, analyze, and interpret health-related data from SMPs. It also involves ensuring that the deployment of such technologies adheres to the ethical principles recognized by the AMIA, including autonomy, beneficence, and nonmaleficence [12]. The ultimate goal is to foster the development and use of AI technologies that enhance health outcomes while minimizing the potential risks and harms that could emerge from their application. Examples of computational methods and models for addressing philosophical ethics include the following: (1) Methods and models focused on the simulation and modeling of ethical dilemmas, such as those using model-based control and Pavlovian mechanisms, are instrumental. These approaches offer valuable insights into the likely outcomes of diverse ethical decisions [118]. (2) Game theory experiments serve as a pivotal means to model and analyze decision-making processes in social contexts, encompassing ethical dilemmas. Notable examples of these experiments include the ultimatum game, the trust game, and the prisoner’s dilemma [119]. (3) The field of data analytics provides methods and models that leverage statistical methods and ML algorithms to scrutinize data. This analysis aims to unearth patterns or insights pertinent to ethical questions or dilemmas [120].

Overall, while methods and models for simulating and modeling ethical dilemmas are capable of effectively representing various scenarios and predicting outcomes, there is a risk that they might oversimplify the complexities inherent in real-world ethics and fail to fully encapsulate the nuances of human ethical reasoning. Although game theory experiments provide insightful perspectives on human behavior in ethical dilemmas, they possess an abstract nature that may limit their practical applicability in realistic situations. Moreover, the efficacy of data analytics methods is heavily dependent on the quality and quantity of the available data. Thus, the application of these methodologies in AI for health care–related interventions on social media should be approached with caution. It is essential to ensure that such applications are in alignment with broader ethical principles.

#### Professional Ethics

In the context of health care interventions via social media, professional ethics refers to a set of guidelines and principles that guide the behavior of health care professionals engaging with social media as part of their practice [121]. These guidelines may cover aspects such as patient privacy; confidentiality; informed consent; and the appropriate use of SMPs for disseminating health information, which includes avoiding conflicts of interest or biased behavior [122]. Algorithms that are designed to detect and flag fraudulent behavior among stakeholders can play a crucial role in identifying potential breaches of professional ethics [123]. Various modeling approaches, such as the living laboratory model, can support the development of health care professional ethics on SMPs [124]. Some researchers call for the development and implementation of local policies at health care organizations to govern the social media activities of health care professionals, highlighting the significant risks associated with the dissemination of information in health care–related social media endeavors [125].

While enforcing professional ethics is vital, it poses challenges, particularly when the methods used may infringe on the autonomy of stakeholders. The strategies mentioned, although essential for upholding ethics, could inadvertently overstep boundaries, thus eliciting concerns regarding the autonomy and privacy of the individuals involved.

#### Legal Ethics

Legal ethics refers to the ethical considerations related to complying with the laws, regulations, and policies surrounding health care data privacy and security. This encompasses safeguarding the confidentiality of patient data, adhering to informed consent and data-sharing agreements, and complying with relevant legal and ethical standards [126,127]. Furthermore, it necessitates ensuring that AI models used in social media interventions for health care are developed and used in conformity with applicable regulations and standards. The existing regulatory and ethical oversight frameworks include the following: (1) the Health Insurance Portability and Accountability Act (HIPAA)—this framework is dedicated to implementing privacy regulations for health care data [145]; (2) the General Data Protection Regulation (GDPR)—it mandates compliance with data protection laws and adherence to other relevant legal and regulatory frameworks governing the use of AI in health care and social media interventions [128]; (3) ethical review boards—advocating for Ethics by Design, this approach involves integrating the services of an ethical review board into the development process of any product within an organization [129].

Both HIPAA and the GDPR are pivotal in the realm of data protection; however, they face intrinsic limitations, with HIPAA being constrained by jurisdictional reach and the GDPR being constrained by the specific subjects it safeguards. The Ethics by Design concept encourages the responsible and ethical development of AI. Nonetheless, this approach could potentially decelerate the innovation process due to the additional layer of review and oversight required during the deployment phase.

#### Other Ethical Considerations

Guttman [130] highlighted a range of ethical concerns tied to health promotion and communication interventions, including issues related to autonomy, equity, the digital divide, consent, and the risk of unintended adverse effects such as stigmatization of certain groups through the use of derogatory terms to describe their medical conditions. The author stressed the importance of identifying and addressing these issues in the context of health care–related communication interventions [130]. This involves safeguarding the privacy and confidentiality of patient data, respecting patient autonomy and consent, and ensuring that the use of SMPs does not harm the patient [131]. Gagnon and Sabus [132] recognized the concerns that health care professionals may have regarding the use of SMPs due to potential factual inaccuracies. Nevertheless, they argued that using social media in health care does not inherently breach ethical principles as long as evidence-based practices are followed, digital professionalism is upheld through controlled information sharing, and the potential benefits of disseminated information outweigh the risks [132].

Bhatia-Lin et al [133] suggested a rubric approach for the ethical use of SMPs in research that is applicable to health care–associated research involving social media surveillance. Wright [63] introduced a framework for assessing the ethical implications of a wide range of technologies whose comprehensiveness renders it a suitable baseline for evaluating the ethical implications of using AI in social media and health care contexts. Various tools, methods, and approaches can aid in ensuring the ethical use of AI within the health care domain on SMPs: (1) data visualization tools—these tools are designed to present complex ethical data in a clear and accessible manner, thus aiding health care professionals and other stakeholders in understanding and making informed decisions [134]; (2) sentiment analysis of social media posts related to health care interventions—this technique identifies ethical issues and concerns, such as biases or stigmatization of certain patient groups, by analyzing the sentiment of social media content [135]; (3) crowdsourcing platforms for ethical feedback—these platforms are developed to gather insights from a wide range of individuals on the ethical implications of AI systems and their recommendations, ensuring the inclusion of diverse perspectives and values (this approach highlights potential ethical concerns that development teams may otherwise overlook [136]); (4) fairness-aware ML algorithms—these algorithms are designed to address and mitigate unfairness in both the training data and the algorithmic decision-making process with the goal of promoting equity [137]; (5) privacy-preserving data analysis—this method emphasizes the protection of sensitive data from unauthorized access while enabling meaningful analysis, thus balancing privacy with utility [138,139]; (6) human-in-the-loop approaches by incorporating human oversight and decision-making into AI systems, these approaches aim to ensure that technology aligns with social values and ethical principles, thereby promoting responsible use [140]; (7) value-sensitive design—this approach focuses on identifying and integrating social values and ethical principles into the design and development of AI systems, thereby promoting their alignment with societal ethics [141].

In summary, each method has distinct applications and limitations. For instance, sentiment analysis of health care–related social media posts is effective in identifying ethical issues such as biases or stigmatization, yet it is susceptible to misinterpretation due to the inherent ambiguity of natural language. On the other hand, human-in-the-loop approaches may introduce subjectivity and diminish the efficiency of automated systems. Consequently, stakeholders involved in applying AI in social media within the health care domain should be cognizant of these methods’ inherent limitations before implementation.

## Discussion

### Principal Findings and Future Research Directions

#### Overview

Health care providers leverage social media to advertise their services, engage with individuals, and cultivate community bonds [146]. SMPs enable medical professionals to interact with patients and gather feedback, thereby enhancing patient care. Moreover, social media acts as a medium for health promotion via peer support and disease awareness initiatives and enabling web-based consultations between physicians and patients [147]. To combat misinformation, implementing rigorous fact-checking measures is imperative for the dissemination of accurate health information. It is also vital to oversee the use of these platforms by health professionals to ensure the protection of patient confidentiality.

The key findings of this study are outlined in the following sections.

#### RQ 1: What Existing Solutions Address FATE in the Context of Health Care on SMPs?

There are 4 identified solutions to FATE in health care discussions on SMPs. First, fairness in this domain is tackled through calibrated, statistical, and intersectional approaches. Calibrated fairness seeks to balance equal opportunities with individual differences, such as language or location. Statistical fairness uses demographic data to prevent biases. Intersectional fairness examines various aspects of an individual’s identity. Second, accountability in health care on SMPs is ensured by adhering to legal standards, incorporating ethical principles into system design, and maintaining technical functionality and privacy, as well as through societal regulation and standardization. These measures include protecting data privacy, preventing discriminatory or unethical use of AI systems, conducting ethical impact assessments, enhancing transparency, involving stakeholders, carrying out audits and evaluations, and holding decision makers responsible. Third, transparency in AI within health care on social media emphasizes the importance of understanding AI systems, including their algorithms, data sources, and decision-making processes. Transparency is vital for comprehending how interventions are crafted, disseminated, and assessed, playing a significant role in identifying and rectifying biases or errors, fostering trust among stakeholders, and improving participation in social media–based health interventions. Fourth, ethics in health care on SMPs focuses on the development of AI technologies that are fair, transparent, and beneficial. This encompasses considerations of privacy, data protection, bias, accountability, and explainability. Upholding professional and social ethics, such as ensuring patient privacy and autonomy, is crucial. The primary aim is to guarantee the ethical use of AI in health care on SMPs while reducing potential risks and adverse effects.

#### RQ 2: How Do the Different Solutions Identified in Response to RQ 1 Compare to Each Other in Terms of Computational Methods, Approaches, and Evaluation Metrics?

The various solutions identified in response to RQ 1 can be compared based on computational methods, approaches, and evaluation metrics. These solutions encompass strategies for achieving calibrated, statistical, and intersectional fairness through a variety of computational methods, including data preprocessing, postprocessing, adversarial training, and decision tree use. Key evaluation metrics for assessing these solutions are equal opportunity and equalized odds. Accountability can be examined from multiple perspectives: legal accountability, achieved through regulatory measures and public-private partnerships; technical accountability, emphasizing logging and auditing; and ethical accountability, focusing on the identification of ethical risks through methods such as ethical impact assessments, value alignment, and stakeholder engagement. Transparency is attainable through several strategies: algorithmic transparency, data transparency, process transparency, and the interpretability and explainability of models. Enhancements in algorithmic transparency can be achieved through feature importance analysis, interpretability techniques for models, and the generation of explanations. Data transparency improvements are facilitated by data visualization, profiling, lineage analysis, and provenance tracking. Process transparency can be bolstered by auditability, monitoring, and adoption of open-source development practices. Although interpretability and explainability remain burgeoning research areas, there is a diverse range of methods for attaining these goals, each suitable for specific contexts. The promotion of ethics in health care on SMPs involves the use of simulation, modeling, data analytics, sentiment analysis, crowdsourcing, and automated systems considering both professional and social ethics.

#### RQ 3: What Is the Strength of the Evidence Supporting the Different Solutions?

The strength of the evidence supporting the solutions is variable and influenced by research quality, methodology, and the statistical significance of the findings. Concepts such as calibrated, statistical, and intersectional fairness are grounded in substantial research. Computational methods, including data preprocessing, adversarial training, and the use of decision trees, are widely adopted, although the extent of evidence supporting their efficacy varies. Evaluation metrics such as equal opportunity and equalized odds rely on well-established statistical measures, but their applicability and effectiveness can differ across studies. Within the ethics domain of health care on SMPs, the principles of privacy protection and bias mitigation are robustly supported by research; however, the evidence for the effectiveness of specific solutions may vary. Techniques such as simulation, modeling, data analytics, and crowdsourcing are commonly used, with their success dependent on the specific application context. Due to the rapidly evolving nature of this field, consulting current and reputable sources is essential for accessing the latest research findings.

The findings from this study contribute to the evolving landscape of AI applications within health care on SMPs by enhancing the understanding of the ethical considerations essential for deploying AI in health care. They delineate practical pathways for leveraging social media to improve patient care and engagement. This study offers insights into achieving fairness in this domain through calibrated, statistical, and intersectional approaches, presenting methodologies that balance personalized care with broader demographic considerations and effectively address biases. It identifies accountability measures such as transparency, documentation, adjudication, stakeholder engagement, logging, and auditing as essential for the design and regulation of AI, ensuring its responsible use in health care contexts. Achieving public transparency presents technical and practical challenges; however, entities involved in AI applications within health care should provide comprehensive reports on decision-making factors, data origins and use, and solid scientific evidence supporting their decisions to stakeholders upon request. Finally, ethical considerations, encompassing philosophical, professional, and legal dimensions, should drive the implementation of the 3 core components of FATE: fairness, accountability, and transparency.

Our study identified several research gaps in AI systems within health care on SMPs. First, primary challenge in the integration of AI and health care on SMPs is the collection and use of data that accurately represent diverse populations without inherent biases. Trustworthy data sets are crucial for training large language models for clinical applications, yet these data sets often lack diversity in key demographics such as age, ethnicity, or medical history. This shortfall can result in AI predictions that disproportionately benefit certain groups. Moreover, the process of obtaining informed consent on SMPs is complicated by the limited understanding users have of how their data might be used for health care research. A common scenario involves the use of patient-generated data from web-based health forums or social media support groups where consent is ambiguously defined, thereby raising ethical and privacy concerns. Second, the operationalization of the broad set of ethical principles defined by the AMIA into a cohesive FATE framework presents significant challenges. The pursuit of a unified approach that addresses the components of FATE simultaneously is hampered by potential conflicts among these principles. For example, increasing transparency by making AI decision-making processes more comprehensible can inadvertently risk patient privacy and system security by exposing sensitive data or proprietary algorithms. Third, the application of FATE principles in real-world health care interventions on SMPs is critically underdocumented. There is a notable absence of comprehensive case studies that detail the implementation, challenges, and outcomes of ethical frameworks in practice. Such documentation is essential for grasping how theoretical ethical considerations are translated into practical impacts and for pinpointing areas that need adjustment when applying these principles. The effectiveness and ethical considerations of AI-driven public health campaigns on platforms such as Twitter and Facebook, for instance, are largely unexplored in a manner that would provide actionable insights into their real-world impact and ethical ramifications. Fourth, the current landscape of evaluating FATE in AI systems, particularly at the intersection of health care and social media, is characterized by a lack of methods that can be universally applied across different models and data types. The specific challenges of the health care domain on SMPs, which include the necessity to analyze diverse data formats in real time, call for the development of model-agnostic tools for ethical assessment. Most existing methods are designed for particular models or data types and do not comprehensively address the wide range of health care applications on social media. Furthermore, there is an absence of a clear strategy for assessing the impact of various AI-assisted interactions between health care and social media domains.

Given the identified gaps, our study proposes 5 research directions. First, research should focus on the development of comprehensive models that integrate the FATE framework with the broader ethical principles outlined by the AMIA. This involves pioneering methodologies that ensure a balanced consideration of all ethical dimensions, aiming to uphold each without compromising the significance or effectiveness of the others. For medical professionals and researchers, this direction represents a shift toward creating AI systems in health care that are both technologically advanced and ethically robust, ensuring equitable and responsible AI use in patient care and data management. Second, investigations are needed into merging computational methods with ethical evaluations to devise sophisticated mathematical formulations capable of quantitatively assessing ethical components in AI applications within health care on SMPs. By developing robust metrics and evaluation frameworks, researchers can bridge the theoretical ethical considerations with practical computational methods. This effort aims to facilitate the integration of ethical principles into the design and evaluation of AI technologies, ensuring that they meet the highest standards of medical ethics and patient care. Third, exploration is required into ethical trade-offs by focusing on understanding and mitigating inherent conflicts between different ethical components within the FATE framework. By systematically examining these trade-offs, research could aim to find innovative solutions that minimize conflicts, such as between transparency and privacy or between fairness and accountability. For the medical and research community, acknowledging and navigating these trade-offs is crucial for the development and implementation of AI systems that are both ethically responsible and effective in achieving health care goals. Fourth, investigation is necessary into the application of FATE principles in real health care interventions on SMPs. This direction seeks to understand the ethical impact of these technologies on users and society. Focusing on the ethical implications of AI-driven health care solutions, from patient engagement strategies to public health campaigns on social media, this research direction aims to ensure that they positively contribute to user well-being and societal health standards. Fifth, a strategic approach should be identified to evaluate the impact of AI-assisted interactions within health care and social media from a FATE perspective. This includes analyzing these interactions to develop universal, model-agnostic metrics that assess the ethical dimensions of AI applications across various platforms. Once established, such metrics could be integrated into social networks, guiding the regulation of AI use in health care on SMPs. For medical professionals and researchers, these metrics would provide a framework for consistently evaluating and ensuring the ethical integrity of AI technologies, promoting safer and more beneficial health care interactions on social media.

### Limitations

The primary limitation of our study stems from the scarcity of comprehensive research that thoroughly explores all dimensions of FATE in the context of AI applications in health care on SMPs. This scarcity reflects not only existing research gaps but also the early stage of scholarly inquiry in this interdisciplinary area. Consequently, our review may not fully encapsulate the complex and multidimensional nature of how FATE intersect and manifest in the deployment of AI within health care settings on social media. This limitation is significant because it suggests that our understanding of FATE issues in this context may rely on an incomplete picture, thus impacting the generalizability of our findings across all potential AI applications in health care on social media.

In addition, identifying the precise population of studies relevant to FATE in AI and health care on SMPs is made more challenging by the heterogeneity and dynamism of SMPs as well as the diversity of AI applications within health care. SMPs are rapidly evolving, introducing new functionalities and altering user interactions, which in turn influences how AI technologies can be applied and examined within these contexts. The challenge of compiling a representative collection of studies that fully encompasses this range contributes to potential gaps in our review, limiting the degree to which our findings can be seen as representative of the field as a whole.

Moreover, the fast-paced advancement of technology, along with the continual evolution of both SMPs and AI, imposes a temporal limitation on our study. Research that was up-to-date at the time of our review may soon become outdated as new technologies emerge and existing ones advance. This swift pace of change implies that the ethical challenges identified today may evolve, new challenges may surface, and previously proposed solutions may become obsolete or less applicable. Therefore, the applicability of our findings is inherently limited by this temporal aspect, underscoring the necessity for ongoing research to continuously refresh our understanding of FATE within AI in health care on SMPs.

### Conclusions

Our review sheds light on the current state of FATE in health care AI as applied to SMPs. It offers a critical analysis of the methodologies, computational techniques, and evaluative strategies used in various studies. By examining the successes and identifying the shortcomings of current practices, this review stimulates further innovation in the field. It challenges existing paradigms on how AI technologies can be both technologically advanced and ethically robust, ensuring fairness, accountability, and transparency in their application.

The practical implications of this work are substantial. First, it guides future research by identifying recent trends and research gaps, suggesting that researchers focus on creating more robust, fair, and ethical AI systems. This includes using diverse data sets that more accurately represent the global population and using evaluation metrics that comprehensively assess the systems’ impacts on all stakeholders. Second, this review underscores the importance of integrating FATE principles throughout the AI system development life cycle, from conceptualization to deployment. For practitioners in health care and technology, this signifies a move toward more inclusive, transparent, and ethically guided development processes. Such a transition not only addresses biases and accountability issues but also boosts patient trust and engagement with AI-driven health care solutions on social media.

Third, the insights from this review are invaluable for policy makers and regulatory bodies, aiding in the creation of nuanced regulations and guidelines that ensure that AI technologies positively contribute to health care outcomes without compromising ethical standards or patient rights. Furthermore, by simplifying complex concepts, this review acts as an educational tool for a broad audience, including health care providers, AI developers, patients, and the general public. Raising awareness about the importance of FATE in health care AI fosters more informed participation in discussions and decision-making regarding AI use in health care, particularly on SMPs.

Ultimately, this study aids in the pursuit of ethical development and deployment of AI systems in health care. By providing an in-depth analysis of the current achievements and future directions for FATE in health care AI on social media, it advocates for the adoption of best practices that balance ethical considerations with technological innovations. The implications of this study extend beyond academia, affecting how AI technologies are conceptualized, developed, and implemented in health care on social media, thereby shaping a future where AI-driven health care solutions are not only effective and innovative but also ethically responsible, equitable, and transparent. This ensures that these technologies serve the best interests of society.

## Appendix

Multimedia Appendix 1 Studies selected for the review.

Multimedia Appendix 2 Fairness evaluation metrics with mathematical formulation.

Multimedia Appendix 3 Accountability evaluation metrics with mathematical formulation.

Multimedia Appendix 4 Transparency evaluation metrics with mathematical formulation.

Multimedia Appendix 5 Ethics evaluation metrics with mathematical formulation.

## Abbreviations

AI: artificial intelligence
AMIA: American Medical Informatics Association
FATE: fairness, accountability, transparency, and ethics
GDPR: General Data Protection Regulation
HIPAA: Health Insurance Portability and Accountability Act
LIME: local interpretable model-agnostic explanations
ML: machine learning
NLP: natural language processing
PDP: partial dependence plot
PRISMA-ScR: Preferred Reporting Items for Systematic Reviews and Meta-Analyses Extension for Scoping Reviews
RQ: research question
SHAP: Shapley additive explanations
SMP: social media platform

## Data Availability

All data generated or analyzed during this study are included in this published paper and its supplementary information files.
