# Supplementary material for: Toward Fairness, Accountability, Transparency, and Ethics in AI for Social Media and Health Care: Scoping Review
Source: JMIR Med Inform. 2024 Apr 3;12:e50048. doi: 10.2196/50048 (PMC11024755; doi:10.2196/50048)
Supplement: Multimedia Appendix 1 [file medinform_v12i1e50048_app1.docx]

Multimedia Appendix 1. Studies selected for the review.

| Study | Fairness | Accountability | Transparency | Ethics |
| --- | --- | --- | --- | --- |
| Mehrabi et al. [1] | ✓ |  |  |  |
| Mashhadi et al. [2] | ✓ |  |  |  |
| Leonelli et al. [3] | ✓ |  |  |  |
| Kington et al. [5] |  | ✓ | ✓ |  |
| Pershad et al. [6] |  |  | ✓ |  |
| Flores and Young [7] |  |  |  | ✓ |
| Pirraglia and Kravitz [8] |  |  |  | ✓ |
| Wieringa [9] |  | ✓ |  |  |
| Hutchinson et al. [10] |  | ✓ |  |  |
| Johnson [11] | ✓ | ✓ | ✓ |  |
| Hagerty and Rubinov [15] |  |  |  | ✓ |
| Gilpin et al. [16] |  |  | ✓ |  |
| Chakraborty et al. [17] | ✓ | ✓ | ✓ |  |
| Carvalho et al. [18] | ✓ | ✓ | ✓ | ✓ |
| Golder et al. [19] |  |  |  | ✓ |
| Bear Don’t Walk et al. [20] | ✓ |  |  |  |
| Attard-Frost et al. [21] | ✓ | ✓ | ✓ | ✓ |
| Adadi and Berrada [22] |  |  | ✓ |  |
| Vian and Kohler [23] |  | ✓ | ✓ |  |
| Saha et al. [26] | ✓ |  |  |  |
| Mehrabi et al. [27] | ✓ |  |  |  |
| Hertweck et al. [28] | ✓ |  |  |  |
| Yao et al. [29] | ✓ |  |  |  |
| Markoulidakis et al. [30] | ✓ |  |  |  |
| Vergeer et al. [31] | ✓ |  |  |  |
| Lagioia et al. [32] | ✓ |  |  |  |
| Saxena et al. [33] | ✓ |  |  |  |
| Park et al. [34] | ✓ |  |  |  |
| Xu et al. [35] | ✓ |  |  |  |
| Tao et al. [36] | ✓ |  |  |  |
| Chouldechova and Roth [37] | ✓ |  |  |  |
| Yao and Huang [38] | ✓ |  |  |  |
| Zhang and Zhou [39] | ✓ |  |  |  |
| Ghassami et al. [40] | ✓ |  |  |  |
| Malawski [41] | ✓ |  |  |  |
| Saleiro et al. [42] | ✓ |  |  |  |
| Bellamy et al. [43] | ✓ |  |  |  |
| Lee et al. [44] | ✓ |  |  |  |
| Bird et al. [45] | ✓ |  |  |  |
| Ghosh et al. [46] | ✓ |  |  |  |
| Zafar et al. [47] | ✓ |  |  |  |
| Chakraborti et al. [48] | ✓ |  |  |  |
| Rosenfeld and Richardson [49] | ✓ |  | ✓ |  |
| Narasimhan et al. [50] | ✓ |  |  |  |
| Kaur et al. [51] |  | ✓ | ✓ |  |
| Bucher et al. [52] |  | ✓ |  |  |
| Kynkäänniemi et al. [53] |  | ✓ |  |  |
| Grandini et al. [54] |  | ✓ |  |  |
| Zaki et al. [55] |  | ✓ |  |  |
| Blacklaws [56] |  | ✓ | ✓ |  |
| Kim et al. [57] |  | ✓ | ✓ |  |
| Dubberley et al. [58] |  | ✓ |  |  |
| Mitchell et al. [59] |  | ✓ |  |  |
| King [60] |  | ✓ |  |  |
| Mittelstadt [61] |  | ✓ |  |  |
| Kass and Faden [62] |  | ✓ | ✓ | ✓ |
| Wright [63] | ✓ | ✓ | ✓ | ✓ |
| Arnold et al. [64] |  | ✓ |  |  |
| Iyer et al. [65] |  | ✓ | ✓ |  |
| Fukuda‐Parr and Gibbons [66] | ✓ | ✓ | ✓ | ✓ |
| Wachter et al. [67] |  | ✓ | ✓ |  |
| Ozga [68] |  | ✓ |  |  |
| Ko et al. [69] |  | ✓ |  |  |
| Raji et al. [70] |  | ✓ |  |  |
| Nushi et al. [71] |  | ✓ | ✓ |  |
| Vesnic-Alujevic et al. [72] |  | ✓ |  | ✓ |
| Kerikmäe and Pärn-Lee [73] |  | ✓ |  | ✓ |
| Reich [74] |  | ✓ | ✓ |  |
| Conway and O’Connor [75] |  | ✓ |  | ✓ |
| Laacke et al. [76] |  |  | ✓ | ✓ |
| Weiskopf et al. [77] |  |  | ✓ |  |
| Crawley et al. [78] |  |  | ✓ |  |
| Zhai et al. [79] |  |  | ✓ |  |
| Weiss et al. [80] |  |  | ✓ |  |
| Burgess et al. [81] |  | ✓ | ✓ |  |
| Diakopoulos and Koliska [82] |  |  | ✓ |  |
| Stellefson et al. [83] |  |  | ✓ | ✓ |
| Valko and Hauskrecht [84] |  |  | ✓ |  |
| Lipton [85] |  |  | ✓ |  |
| Slack et al. [86] |  |  | ✓ |  |
| Stepin et al. [87] |  |  | ✓ |  |
| Bertino et al. [88] |  | ✓ | ✓ |  |
| He et al. [89] |  |  | ✓ |  |
| Li [90] |  |  | ✓ |  |
| Azeroual et al. [91] |  |  | ✓ |  |
| Tang et al. [92] |  | ✓ | ✓ |  |
| Leslie [93] |  | ✓ | ✓ | ✓ |
| Shneiderman [94] |  |  | ✓ | ✓ |
| Brundage et al. [95] |  | ✓ | ✓ |  |
| Janssen et al. [96] |  |  | ✓ |  |
| Paredes et al. [97] |  |  | ✓ |  |
| Amann et al. [98] |  |  | ✓ |  |
| Arrieta et al. [99] |  |  | ✓ |  |
| Xiong et al. [100] |  |  | ✓ |  |
| Zafar and Khan [101] |  |  | ✓ |  |
| Lundberg and Lee [102] |  |  | ✓ |  |
| Sokol and Flach [103] |  |  | ✓ |  |
| Tjoa and Guan [104] |  |  | ✓ |  |
| Vig [105] |  |  | ✓ |  |
| Fan et al. [106] |  | ✓ | ✓ |  |
| Murdoch et al. [107] |  |  | ✓ |  |
| Leikas et al. [108] |  | ✓ | ✓ | ✓ |
| Latonero [109] |  |  | ✓ | ✓ |
| Aiello et al. [110] |  |  |  | ✓ |
| Olteanu et al. [111] | ✓ |  |  | ✓ |
| Dixon et al. [112] | ✓ |  |  | ✓ |
| Feldman et al. [113] | ✓ |  |  | ✓ |
| Mendes et al. [114] |  |  |  | ✓ |
| Datta et al. [115] |  |  | ✓ | ✓ |
| Kazim and Koshiyama [116] |  |  |  | ✓ |
| Nebeker et al. [117] |  | ✓ |  | ✓ |
| Crockett [118] |  |  |  | ✓ |
| Colman [119] |  |  |  | ✓ |
| Someh et al. [120] |  |  |  | ✓ |
| Ventola [121] |  |  |  | ✓ |
| Ponce et al. [122] |  |  | ✓ | ✓ |
| Drabiak and Wolfson [123] |  |  | ✓ | ✓ |
| Neville and Waylen [124] |  |  |  | ✓ |
| Ennis-O-Connor and Mannion [125] |  |  |  | ✓ |
| Garg and Shrigiriwar [126] |  |  |  | ✓ |
| Kalkman et al. [127] |  | ✓ | ✓ | ✓ |
| Sharma [128] |  |  | ✓ | ✓ |
| Leidner and Plachouras [129] |  |  |  | ✓ |
| Guttman [130] |  |  |  | ✓ |
| Denecke et al. [131] |  |  |  | ✓ |
| Gagnon and Sabus [132] |  |  |  | ✓ |
| Bhatia-Lin et al. [133] |  | ✓ |  | ✓ |
| Davis [134] |  |  |  | ✓ |
| Livingston et al. [135] |  |  |  | ✓ |
| Jakesch et al. [136] | ✓ |  |  | ✓ |
| Pastaltzidis et al. [137] | ✓ |  |  | ✓ |
| Keshk et al. [138] |  |  | ✓ | ✓ |
| Kayaalp [139] |  |  | ✓ | ✓ |
| Enarsson et al. [140] |  |  |  | ✓ |
| Umbrello and van de Poel [141] | ✓ |  |  | ✓ |
