# Supplementary material for: Toward Fairness, Accountability, Transparency, and Ethics in AI for Social Media and Health Care: Scoping Review
Source: JMIR Med Inform. 2024 Apr 3;12:e50048. doi: 10.2196/50048 (PMC11024755; doi:10.2196/50048)
Supplement: Multimedia Appendix 2 [file medinform_v12i1e50048_app2.docx]

Multimedia Appendix 2. Fairness evaluation metrics with mathematical formulation. FP = False Positive, FN = False Negative, TP = True Positive, TN = True Negative

| **Metric** | **Formula** | **Description** |
| --- | --- | --- |
| Equality of Opportunity Difference (EqOpp) [27] | $\text{EqOpp}\left( \hat{y},a,y \right)=\max_{a_{i},a_{j}} \left\vert P\left( \hat{y}=1 \vert a=a_{i},y=1 \right)-P\left( \hat{y}=1 \vert a=a_{j},y=1 \right) \right\vert$  where:  $a\in\{a_{1},\ldots,a_{l}\}$is a discrete variable with *l* possible values and denotes the sensitive or protected attributes with respect to which we want to be fair.  $y \in\{0, 1\}$is the true label.  $\hat{y}$is the binary outcome of the model. | Measures chances of receiving favorable outcomes for the advantaged and disadvantaged groups, given that the actual outcome is positive. |
| Equalized Odds Difference (EqOdd) [27] | $\text{EqOdd}\left( \hat{y},a,y \right)=\max_{a_{i},a_{j}} \max_{y\in\{0,1\}} \left\vert P\left( \hat{y}=1 \vert a=a_{i},y=y \right)-P\left( \hat{y}=1 \vert a=a_{j},y=y \right) \right\vert$  where:  $a\in\{a_{1},\ldots,a_{l}\}$is a discrete variable with *l* possible values and denotes the sensitive or protected attributes with respect to which we want to be fair.  $y \in\{0, 1\}$is the true label.  $\hat{y}$is the binary outcome of the model. | Facilitates ensuring equal accuracy of the model’s predictions irrespective of group membership. |
| Statistical Parity Difference (SPD): [27] | $\text{SPD}\left( \hat{y},a \right)=\max_{a_{i},a_{j}} \left\vert P\left( \hat{y}=1 \vert a=a_{i} \right)-P\left( \hat{y}=1 \vert a=a_{j} \right) \right\vert$  where:  $a\in\{a_{1},\ldots,a_{l}\}$is a discrete variable with *l* possible values and denotes the sensitive or protected attributes with respect to which we want to be fair.  $y \in\{0, 1\}$is the true label.  $\hat{y}$is the binary outcome of the model. | Measures the difference in the probability of receiving a favorable outcome between groups, focusing on ensuring that outcomes are independent of group membership. |
| Statistical Parity [28] | $\forall a\in A,P(Y=1\mid A=a)=P(Y=1)$  where:  *A* represents the set of all possible values of a particular sensitive attribute | The proportion of favorable outcomes is the same for all groups |
| Accuracy [142] | $\frac{\left( TP + TN \right)}{Total Population}$ | The proportion of all predictions that are correct. |
| False Positive Rate Difference (FPRD) [29] | $FPRD = \sum_{t\in T} \left\vert FPR-FPR_{t} \right\vert$  where:  $\mathrm{FP}R_{t}$ is the false positive rate computed upon the subset of samples that contain the term *t* | Compares the False Positive Rates between groups, showing disparities in the rates at which negative instances are incorrectly classified as positive. |
| True Positive Rate (TPR) [143] | $\frac{FN}{\left( FN + TP \right)}$ | The proportion of positive instances that are correctly classified as positive. Also known as sensitivity or recall. |
| True Negative Rate (TNR) [143] | $\frac{TN}{\left( TN + FP \right)}$ | The proportion of negative instances that are correctly classified as negative. Also known as specificity. |
| False Discovery Rate (FDR) [30] | $\frac{FP}{\left( FP + TP \right)}$ | The proportion of positive predictions that are false |
| False Omission Rate (FOR) [30] | $\frac{FN}{\left( FN + TN \right)}$ | The proportion of negative predictions that are false. |
| Positive Likelihood Ratio (LR+) [31] | $\frac{TPR}{FPR}$ | Indicates how much more likely a positive result is to occur when the condition is present than when it is absent. |
| Negative Likelihood Ratio (LR-) [31] | $\frac{FNR}{TNR}$ | Indicates how much more likely a negative result is to occur when the condition is absent than when it is present. |
| Group Parity [32] | $\frac{\#TP\_for\_groupA}{\#actual\_positives\_for\_group\_A}-\frac{\#TP\_for\_groupB}{\#actual\_positives\_for\_groupB}$ | Measures whether the AI system treats different groups fairly |
