# Supplementary material for: Toward Fairness, Accountability, Transparency, and Ethics in AI for Social Media and Health Care: Scoping Review
Source: JMIR Med Inform. 2024 Apr 3;12:e50048. doi: 10.2196/50048 (PMC11024755; doi:10.2196/50048)
Supplement: Multimedia Appendix 3 [file medinform_v12i1e50048_app3.docx]

Multimedia Appendix 3. Accountability evaluation metrics with mathematical formulation. Accuracy, False Positive Rate, and False Negative Rate metrics are also suitable for evaluating accountability in AI systems, as discussed in (Multimedia Appendix 2). FP = False Positive, FN = False Negative, TP = True Positive, TN = True Negative

| **Metric** | **Formula** | **Description** |
| --- | --- | --- |
| Trustworthy Explainability Acceptance [51] | $T_{wA}=\left( \sum A_{e}T_{e} \right)/E$  where:  $A_{e}=1-\sqrt{\frac{\sum_{i=1}^{n} \left( X_{i}-Y_{i} \right)^{2}}{n}}$  $A_{e}$is explainability acceptance by expert *e*, lies between [0,1].  *X* and *Y* are two *n*-dimensional explanations.  *E* is the total number of experts.  $T_{e}$is the trust of expert *e* | Measures acceptance by quantifying the distance between the explanations generated by the AI system and the reasoning provided by the experts based on their expertise and experience. |
| Embedding Consistency Score [52] | $S\left( X,Y \right)=d_{A}\left( \hat{A_{X}}\left( X \right),\hat{A_{Y}}\left( Y \right) \right)=\left. \vert\left( \hat{A_{X}}\left( X \right)-\hat{A_{Y}}\left( Y \right) \right)^{T}W_{A} \right.\vert_{2}$  where:  $\hat{A_{X}}\left( X \right),\hat{A_{Y}}\left( Y \right)$are embeddings for modalities X and Y (modalities can be represented by features).  $W_{A}$is Mahalanobis mapping. | Can facilitate measuring consistency across models and representations |
| Precision [53] | $\frac{TP}{TP+FP}$ | The proportion of true positive predictions among all positive predictions. |
| Recall (Sensitivity) [53] | $\frac{TP}{TP+FN}$ | The proportion of true positive predictions among all actual positive instances. |
| Specificity [144] | $\frac{TN}{TN+FP}$ | The proportion of true negative predictions among all actual negative instances. |
| F1 Score [54] | $2*\frac{Precision*Recall}{Precision+Recall}$ | The harmonic mean of precision and recall. |
