# Supplementary material for: Toward Fairness, Accountability, Transparency, and Ethics in AI for Social Media and Health Care: Scoping Review
Source: JMIR Med Inform. 2024 Apr 3;12:e50048. doi: 10.2196/50048 (PMC11024755; doi:10.2196/50048)
Supplement: Multimedia Appendix 4 [file medinform_v12i1e50048_app4.docx]

Multimedia Appendix 4. Transparency evaluation metrics with mathematical formulation.

| **Metric** | **Formula** | **Description** |
| --- | --- | --- |
| Completeness [77] | $I=\frac{2}{n}+\frac{n-2}{n}\left( 1-\sqrt{\left( n-1 \right)\cdot\text{Var}\left( g_{t};i=1,\ldots,n-1 \right)} \right)$  where:  $g_{t}=\frac{x_{i+1}-x_{i}}{x_{n}-x_{1}}$ is a normalized difference between contiguous data points  *n* is the total number of data points  $x_{n}$ is the *n-th* data point | $I$ represents the average amount of information provided by each data point taking variability between these points into account |
| Timeliness [78] | $\text{Timeliness}=T_{\text{Detect}}-T_{\text{Start}}$  where:  $T_{\text{Detect}}$ is time when an accident was detected  $T_{\text{Start}}$ is time when an accident took place (started) | The time interval between two milestones: shows how soon an incident was detected |
| Relevance [79], broad definition | $\text{Relevance}=\frac{D_{\text{rel}}}{D_{\text{total}}}$  where:  $D_{\text{rel}}$ is the number of relevant data points  $D_{\text{total}}$ is total number of data points | The extent to which data is applicable to the problem at hand |
| Accessibility [80], broad definition | $\text{Accessibility}=\frac{D_{o}}{D_{\text{total}}}$  where:  $D_{\text{o}}$ is the number of data points that can be easily obtained  $D_{\text{total}}$ is total number of data points | The extent to which data is easy to obtain and use |
| Data Provenance [81], broad definition | $\text{Provenance}=\frac{D_{p}}{D_{\text{total}}}$  where:  $D_{p}$ is the number of data points for which we track the origin  $D_{\text{total}}$ is total number of data points | Provenance involves tracking the origin, processing history, and movement of data throughout the AI system. |
