# Supplementary material for: Toward Fairness, Accountability, Transparency, and Ethics in AI for Social Media and Health Care: Scoping Review
Source: JMIR Med Inform. 2024 Apr 3;12:e50048. doi: 10.2196/50048 (PMC11024755; doi:10.2196/50048)
Supplement: Multimedia Appendix 5 [file medinform_v12i1e50048_app5.docx]

Multimedia Appendix 5. Ethics evaluation metrics with mathematical formulation. FP = False Positive, FN = False Negative, TP = True Positive, TN = True Negative.

| **Metric** | **Formula** | **Description** |
| --- | --- | --- |
| Pinned AUC Equality Difference [112] | $\text{Pinned AUC Equality Difference}=\sum_{t\in T} \left\vert\text{AUC}-\text{pAUC}_{t} \right\vert$  where AUCt is a per-term (*t*) pinned AUC  AUC is AUC on the aggregated data over all identity terms | Measures variance between performance on individual terms. Can be employed for assessing bias. |
| Disparate Impact [113] | $\frac{P\left( positive outcome \vert Group A \right)}{P\left( positive outcome \vert GroupB \right)}$ | Measures whether an AI system is treating different groups of people unfairly. |
| Privacy [114], broad definition | $\text{Privacy}=1-P\left( \text{id} \right)$  where:  $P\left( \text{id} \right)$ is the probability to identify user from depersonalized data | Measures the extent to which an AI system is protecting the privacy of individuals. |
| Quantitative Input Influence for Group Disparity [115] | $\iota_{\text{disp}}^{\mathcal{Y}}\left( i \right)=Q_{\text{disp}}^{\mathcal{Y}}\left( X \right)-Q_{\text{disp}}^{\mathcal{Y}}\left( X_{-i}U_{i} \right)$  where:  $Q_{\text{disp}}^{Y}\left( \cdot\right)=\left\vert E\left[ c\left( \cdot\right)=1 \mid X\in\mathcal{Y} \right]-E\left[ c\left( \cdot\right)=1 \mid X\notin\mathcal{Y} \right] \right\vert$ is a difference in classification rates.  $X$ is original distribution.  $X_{-i}U_{i}$ is intervened distribution.  $\mathcal{Y}$ is a set representing a protected group.  $c\left( \cdot\right)$ is a classifier (the dot represents an argument). | Measures the degree of influence of inputs on outputs  of systems. Can be used to detect algorithmic discrimination |
